# Supplementary material for: VH-Based Mini Q-Body: A Novel Quench-Based Immunosensor
Source: Sensors (Basel). 2023 Feb 17;23(4):2251. doi: 10.3390/s23042251 (PMC9960136; doi:10.3390/s23042251)
Supplement: Supplementary file 1 [file sensors-23-02251-s001.zip › sensors-2226361-supplementary.pdf]

Supplementary Information for

V_H-Based Mini Q-Body: A Novel Quench-Based Immunosensor

Jinhua Dong [1,2,3,†], Bhagat Banwait [4,†], Hiroshi Ueda [2,‡] and Peter Kristensen [5,*]

1 School of Rehabilitation Sciences and Engineering, University of Health and Rehabilitation Sciences, Qingdao 266071, China
2 Laboratory for Chemistry and Life Science, Institute of Innovative Research, Tokyo Institute of Technology, Yokohama 226-8503, Japan
3 International Research Frontiers Initiative, Tokyo Institute of Technology, Yokohama 226-8503, Japan
4 Department of Engineering, Aarhus University, 8000 Aarhus, Denmark
5 Department of Chemistry and Bioscience, Aalborg University, 9220 Aalborg, Denmark
* Correspondence: pk@bio.aau.dk; Tel.: +45-2036-9097
† These authors contributed equally to this work.
‡ Deceased.

Table S1. Primers used in this study.

Primer name	Sequence (5′-3′)
FWR-C3-Age	GGCCACCGGTGAAGTTCAGCTGCTGGAAAG
REV-C3-BamHI	CCATGGATCCGCCGTGGTGGTGGTGGTGGTGCT
W36toFback	GATGAAGATATGGGCTTTGTGCGCCAGGCA
W36toFbfor	CAGGTGCCTGGCGCACAAAGCCCATATCTTCATC
W47toFback	GTAAAGGCCTGGAATTTGTGAGCTCCATTTATGGC
W47toFfor	GCCATAAATGGAGCTCACAAATTCCAGGCCTTTAC
W103toFback	AACGTTTTCGACTACTTTGGCCAGGGTACCCTGGTG
W103toFfor	CACCAGGGTACCCTGGCCAAAGTAGTCGAAAACGTT
T7 Promoter	TAATACGACTCACTATAGGG
T7 Terminator	GCTAGTTATTGCTCAGCGG

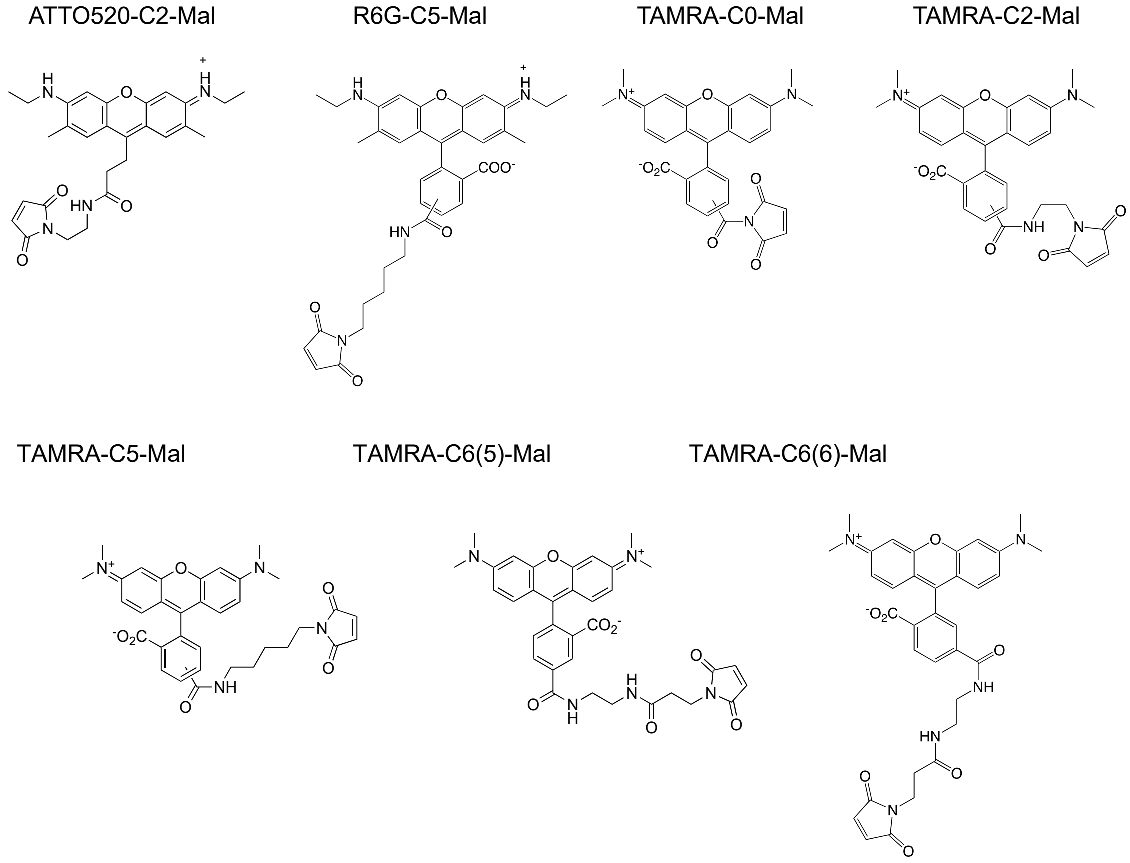

Figure S1. Structures of fluorescent dyes used in this study.

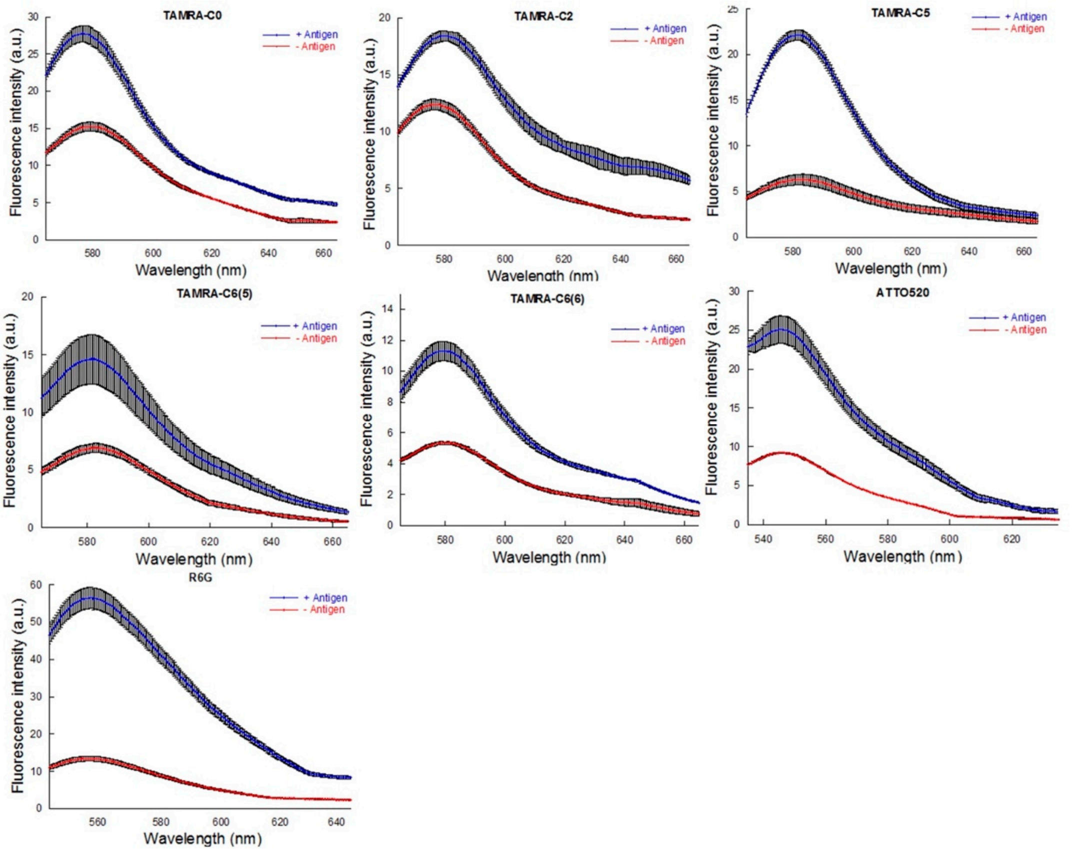

Figure S2. Antigen-dependent fluorescence response of C3 mini Q-bodies in the presence and absence of antigen. The black shaded area represents ±1 SD (n = 3).

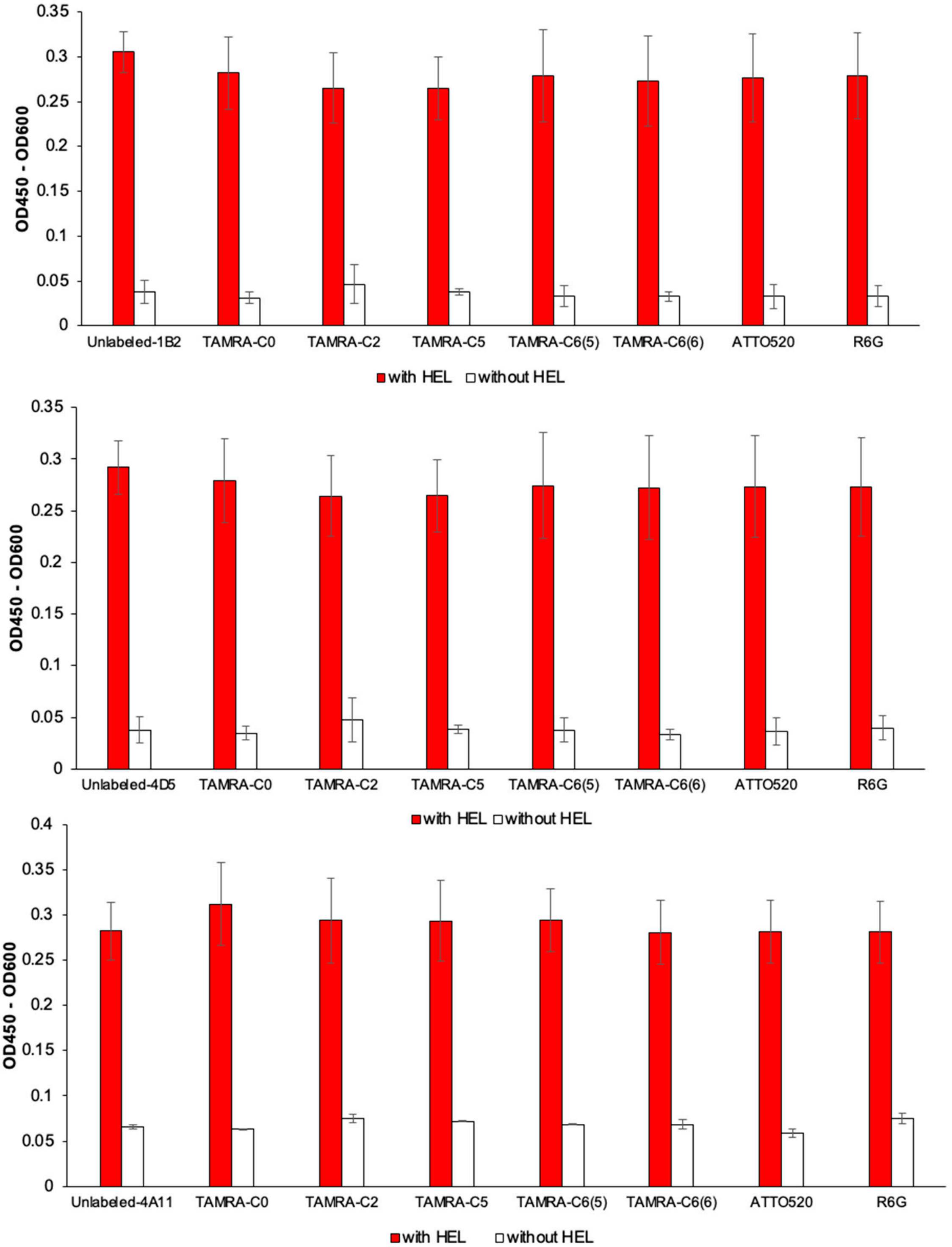

Figure S3. Specific binding of unlabeled 1B2, 4D5, 4A11 and their derived mini Q-bodies to HEL, measured by ELISA. The colored bars indicate the signal measured while the white bars stand for the signal in the absence of antigen. Error bars show the standard deviation (n = 3).
